# Interleukin-6 mediates delirium-like phenotypes in a murine model of urinary tract infection

**DOI:** 10.1186/s12974-021-02304-x

**Published:** 2021-10-28

**Authors:** Mohammad Harun Rashid, Nicklaus A. Sparrow, Faizan Anwar, Gena Guidry, Ambart E. Covarrubias, Haoming Pang, Chandrakumar Bogguri, S. Ananth Karumanchi, Shouri Lahiri

**Affiliations:** 1 Department of Neurology, Cedars-Sinai Medical Center, Los Angeles, CA USA; 2 Department of Medicine, Cedars-Sinai Medical Center, Los Angeles, CA USA; 3 Department of Pathology, Cedars-Sinai Medical Center, Los Angeles, CA USA; 4 Departments of Neurology, Neurosurgery, and Biomedical Sciences, Cedars-Sinai Medical Center, 8700 Beverly Blvd., Los Angeles, CA 90048 USA

**Keywords:** Delirium, UTI, Neuron, Neuroinflammation, IL-6, IL-6 inhibition

## Abstract

**Background:**

Urinary tract infection (UTI) is frequently implicated as a precipitant of delirium, which refers to an acute confusional state that is associated with high mortality, increased length of stay, and long-term cognitive decline. The pathogenesis of delirium is thought to involve cytokine-mediated neuronal dysfunction of the frontal cortex and hippocampus. We hypothesized that systemic IL-6 inhibition would mitigate delirium-like phenotypes in a mouse model of UTI.

**Methods:**

C57/BL6 mice were randomized to either: (1) non-UTI control, (2) UTI, and (3) UTI + anti-IL-6 antibody. UTI was induced by transurethral inoculation of 1 × 10^8^ *Escherichia coli*. Frontal cortex and hippocampus-mediated behaviors were evaluated using functional testing and corresponding structural changes were evaluated via quantification of neuronal cleaved caspase-3 (CC3) by immunohistochemistry and western blot. IL-6 in the brain and plasma were evaluated using immunohistochemistry, ELISA, and RT-PCR.

**Results:**

Compared to non-UTI control mice, mice with UTI demonstrated significantly greater impairments in frontal and hippocampus-mediated behaviors, specifically increased thigmotaxis in Open Field (*p* < 0.05) and reduced spontaneous alternations in Y-maze (*p* < 0.01), while treatment of UTI mice with systemic anti-IL-6 fully reversed these functional impairments. These behavioral impairments correlated with frontal and hippocampal neuronal CC3 changes, with significantly increased frontal and hippocampal CC3 in UTI mice compared to non-UTI controls (*p* < 0.0001), and full reversal of UTI-induced CC3 neuronal changes following treatment with systemic anti-IL-6 antibody (*p* < 0.0001). Plasma IL-6 was significantly elevated in UTI mice compared to non-UTI controls (*p* < 0.01) and there were positive and significant correlations between plasma IL-6 and frontal CC3 (*r*^2^ = 0.5087/*p* = 0.0028) and frontal IL-6 and CC3 (*r*^2^ = 0.2653, *p* < 0.0001).

**Conclusions:**

These data provide evidence for a role for IL-6 in mediating delirium-like phenotypes in a mouse model of UTI. These findings provide pre-clinical justification for clinical investigations of IL-6 inhibitors to treat UTI-induced delirium.

**Supplementary Information:**

The online version contains supplementary material available at 10.1186/s12974-021-02304-x.

## Introduction

Urinary tract infection (UTI) is a common condition that affects an estimated 150 million individuals worldwide per year [1]. Delirium complicates recovery in approximately one-third of patients with UTI and is characterized by a constellation of symptoms that reflect dysfunction of the frontal cortex and hippocampus, including psychomotor agitation, inattentiveness, and short-term memory impairment [2–6].

Although upregulation of systemic interleukin-6 (IL-6) and other inflammatory cytokines have been reported in systemic infection models, it remains unknown whether these cytokines contribute to the pathogenesis of delirium. In a mouse model of non-infectious acute lung injury, we recently demonstrated that systemic IL-6 inhibition reverses delirium-like neuronal changes in the frontal cortex and hippocampus, suggesting a key role for IL-6 in mediating delirium-like structural phenotypes [7]. Accordingly, in this study, we sought to examine whether UTI produces symptoms that resemble delirium, and to study mechanisms by which this may occur, by characterizing the functional and structural role of IL-6 in a mouse model of UTI.

## Methods

### Mice and power analysis

#### Model development/hypothesis generation

As UTI occurs most commonly in women [8], we used female mice only. Eighteen 6–7-month-old female mice were used to develop the model of UTI-induced delirium. Mice were randomized to receive either transurethral inoculation of sterile PBS-treatment with normal saline (control group, *n* = 9), or transurethral inoculation of *Escherichia coli* (*E. coli*)-treatment with normal saline (UTI group, *n* = 9). Detailed methodology is shown below.

#### Hypothesis validation

Based on preliminary immunohistochemical analysis using cleaved caspase-3 (CC3), the greatest group standard deviation and mean difference were 0.0869 and 0.1876, respectively. Using these values, a power analysis with one-way ANOVA and Tukey’s post-hoc test yielded greater than 95% power at the 0.05 significance level for an effect size of 1.1317 with a minimum of *n* = 8/group.

A total of 105 female C57BL/6 mice, aged 6–7 months (Jackson Laboratory, Bar Harbor, ME), were used in this study. To examine the effects of systemic IL-6 inhibition on functional and structural delirium-like phenotypes, mice were randomized to one of the following groups: (1) transurethral inoculation of sterile PBS-treated with normal saline (control group, *n* = 16), (2) transurethral inoculation of *E. coli*-treated with normal saline (UTI group, *n* = 20), and (3) transurethral inoculation of *E. coli*-treated with IL-6 function-blocking monoclonal antibody (UTI + α-IL-6 antibody group, *n* = 19).

### UTI model

Static cultures of *E. coli* (Migula) Castellani and Chalmers (ATCC® 700928™) strain were grown for 24 h at 37 °C. Bacterial cultures were harvested by centrifugation (4000 rpm for 10 min at 4 °C) and resuspended in sterile endotoxin-free phosphate-buffered saline (PBS; PromoCell, Heidelberg, Germany) at a concentration of 1 × 10^9^ colony forming unit/mL (cfu/mL). Mice were inoculated transurethrally as previously described [9]. Briefly, 6–7-month-old female C57BL/6 mice were quarantined for a minimum of 1 week prior to inoculation and allowed food and water ad libitum. Mice were anesthetized with isoflurane USP (Clipper Distribution Company LLC, St. Joseph, MO) delivered by a vaporizer (Vet Equip Inc., Pleasanton, CA) set at 2% isoflurane and 0.8 L of oxygen per minute. The inoculum was delivered via a 1-ml syringe connected to a 1/2-in., 30-gauge needle. The catheters were prepared with sterile 0.61-mm (internal diameter) polyethylene tubing (PE 10, Becton, Dickinson, Sparks, MD). The needle was inserted into the catheter, and 100 µL of inoculum containing 1 × 10^8^ cfu of *E. coli* was delivered. Control mice received 100 µL of sterile normal saline as inoculum. Urine samples were collected and processed daily for bacterial enumeration and determination of the intensity of pyuria. After 3 days, mice were euthanized by perfusion while deeply anesthetized with a combination of ketamine and dexmedetomidine followed by a physical method for tissue collection, and the brain was aseptically removed.

### IL-6 inhibition

IL-6 signaling was inhibited by systemic administration of monoclonal antibody which binds IL-6 peptide (α-IL-6) (Bio X Cell α-IL-6, clone MP5-20F3). Each antibody-treated mouse received 200 µg of antibody in a 400µL solution of normal saline as an intraperitoneal injection once daily (total 3 doses). The average dose was 7.6 mg/kg. Control mice and UTI mice received 400µL of normal saline only.

### Behavioral testing

Locomotor activity and anxiety-related emotional behaviors were evaluated by placing mice individually into the center of a clear Plexiglas (40 × 40 × 40 cm) open-field arena and allowing the mouse to explore for 45 min. Activity in the open-field was recorded by a computer-operated camera system (Stoelting Digital USB 2.0 CMOS Camera). Total distance (locomotor activity), movement time (in seconds), movement speed (cm/s), and center distance (the distance traveled in the center of the arena) were analyzed by ANY-maze Video Tracking Software version 6.34 (Stoelting Co., Wood Dale, IL, USA). The open field was divided into a 20 × 20 cm central zone and a surrounding border zone during the analysis. The center distance was divided by the total distance to obtain a center distance–total distance ratio. The center distance–total distance ratio can be used as an index of anxiety-related responses. Data were collected in 5 min intervals over the 45 min test session. For Y-maze, each arm of the maze was labeled as either A, B, or C. In each session, the animal is placed in arm A and allowed to explore the three arms for 5 min. Activity in the Y-maze was recorded by a computer-operated video recording system. Number of arm entries are scored from the recorded video file to calculate the percent alternation. The alternation percentage is calculated by dividing the number of alternations by number of possible triads × 100. The maze is cleaned with Virkon solution between animals to eliminate odor traces.

### Brain isolation and treatment

Mice were deeply anesthetized and perfused with room temperature PBS with 0.5 mM ethylenediaminetetraacetic acid (10 mL). Right hemispheres were collected and fixed by submerging in ice-cold PBS buffered 4% paraformaldehyde (Electron Microscopy Sciences) for 30 min, and then cryo-protected in 2% paraformaldehyde + 30% sucrose at 4 °C for 24–48 h. Free-floating, 30-μm-thick coronal brain cryosections were prepared and stored at 4 °C in PBS + 0.02% sodium azide until staining.

### Immunohistochemistry and microscopy

Sections were affixed to slides by air drying and subjected to heat-induced epitope retrieval for 10 min in antigen-retrieval solution (pH 6.0; Invitrogen) before permeabilization/blocking in 5% BSA + 0.25% Triton X-100 in PBS for 1 h at room temperature. Sections were then incubated at 4 °C overnight with primary antibodies diluted in 1% BSA + 0.01% Triton X-100 in PBS (Ab Diluent). After washing, sections were incubated with a combination of the appropriate secondary antibody (Alexa Fluor Plus conjugated; Invitrogen) diluted to 4 µg/mL in Ab Diluent for 1 h at room temperature. After washing, sections were incubated in 0.05% Sudan black B in 70% ethanol for 10 min to reduce tissue autofluorescence. Sections were mounted using ProLong Glass with DAPI (Invitrogen, Carlsbad, CA, USA). Negative controls were processed using the same protocol with the omission of the primary antibody to assess non-specific labeling. A Carl Zeiss AxioImager Z.2 epi-fluorescence microscope equipped with standard filter sets/mercury arch lamp, an Apotome 2.0, and an Axiocam HRm camera controlled by Zen Blue Pro (version 2.3) software was used to acquire and process images. Images of damage marker (e.g., CC3) staining were acquired with a × 10 objective (NA 0.3, Zeiss) as a 5 × 5 tiled image that encompassed the compass and hippocampus of each section. Images of IL-6 staining were acquired with the Apotome 2.0 and a × 20 objective (NA 0.8, Zeiss) as a single field, 8 µm z-stacks (1 µm interval), and were analyzed and displayed as maximum intensity projections. All acquisition and display settings are the same between groups and settings were optimized using the UTI group. All images within a figure are set to the same scale.

### Image analysis

Fiji (ImageJ v. 1.53c) software was used for image analysis and semi-quantitation. Prism 9.0.0 (GraphPad) was used for statistical analysis. Three coronal sections containing the cortex and hippocampus were analyzed (one ventral, one mid, and one dorsal) per animal. For damage marker analysis, two different regions of interest (ROIs) were drawn on tiled images of sections: a ROI around the cortex (with an average area of 315 µm^2^), or a ROI encompassing the entire hippocampus. A threshold was set to exclude background pixels per ROI based on the pixel intensity histogram, and the number of positive pixels was measured and then expressed as percent area of the ROI. For cytokine analysis, a single field z-stack projection from the cortex was analyzed per section. Background pixels were excluded per field based on the pixel intensity histogram and the intensity of the remaining pixels was used to calculate percent area. Values for each protein from the triplicate sections were averaged to yield one number per animal. Analysis was performed by assessors blinded to group allocation.

### Western blotting

Brain tissues were homogenized for protein extraction using Pierce RIPA buffer (Thermo Scientific, USA) with protease and phosphatase inhibitors. Following 30 min of incubation on ice, the lysate was centrifuged at 15,000 rpm for 15 min in 4 °C. Protein concentrations were estimated with Pierce, BCA protein assay kit (Thermo Scientific, USA). Equal amounts of proteins (50 μg) were separated on a SDS 4–12%-polyacrylamide gel, and then transferred to a nitrocellulose membrane (Invitrogen, Carlsbad, CA, USA). Blots were blocked for 1 h at room temperature with 5% (w/v) BSA in PBST (PBS + 0.01% TX-100). Then the membrane was incubated at 4 °C with specific primary antibodies overnight. Antibodies against CC3 (1:600 dilution, Cell Signaling Technology, catalog #9664, Danvers, MA, USA) and β-actin (1:50,000 dilution, catalog # MA5-15739-HRP, MA, USA) were used in the study. After 3 wash steps with PBST, the blot was then incubated with the corresponding horseradish peroxidase‐conjugated secondary antibody (1:30,000). The blot was developed using a Pierce Super Signal West Pico Chemiluminescent substrate kit (Thermo Scientific, USA). Western blot images were acquired by iBright Western Blot Imaging Systems (Invitrogen, Carlsbad, CA, USA) and analyzed by iBright Analysis Software Version 3.1.3.

### Urine collection

The animals were manually restrained by gentle grasping on either side of the neck and drawing up the loose skin, trapping the fold gently between the finger and thumb to ensure that the weight of the animal is supported. To collect urine, the mice were held over an aluminum foil and allowed to voluntarily void their bladders. Urine was collected into a sterile tube for further processing.

### Quantification of plasma IL-6

ELISA was carried out per manufacturer’s instructions (R&D Systems, Quantikine™ ELISA Mouse IL-6, Catalog number M6000B, Minneapolis, MN). Reagent, samples, and standard solutions were prepared as directed and allowed to come to room temperature before use. A standard curve with known IL-6 concentrations (7.8–500 pg/mL) was run in parallel. Data from the technical replicate wells were averaged to obtain a mean value per plasma rich protein sample diluted at 1:2 before use. A positive control of mouse IL-6 sample of known concentration was also added. Plates were manually washed and aspirated between steps to lower background noise. Once the plate was complete it was read at a wavelength of 450 nm using Bio-Rad xMark™ Microplate Absorbance Spectrophotometer, with a correction wavelength of 540 nm.

### Secondary measure: quantification of cortical IL-6

The QUANTERIX Mouse IL-6 Discovery assay (Quanterix Corporation, Billerica, MA) was performed using a sensitive, fully automated ELISA platform, Simoa HD-X Analyzer. Each well is loaded with 350ul of diluted (1:5) cortical protein sample. Each Quanterix Mouse IL-6 Discovery kit (Cat#102109 Quanterix) contains eight calibrators, two controls, sample diluent, Bead, Detector, SBG (streptavidin beta galactosidase) and RGP (fluorogenic β-galactosidase substrate resorufin) reagents. All the reagents are prepared and set up in Simoa HD-X system according to manufacture protocol. During the assay, each sample was mixed with magnetic beads conjugated with capture antibody, and subsequently mixed with the detector antibody, SBG, and RGP. Each sample was run in duplicate, and concentration is calculated based on Average Enzyme per Bead (AEB). Any sample with the coefficient of variation (CV) higher than 20% was repeated with appropriate calibrators and controls.

### Quantitative real-time PCR for cortical IL-6 mRNA

Total RNA was isolated from frozen left-brain hemispheres samples using Trizol reagent (Invitrogen) following manufacturer's protocol. RNA concentration was measured using nanodrop spectrophotometer, and integrity of RNA was determined using denatured agarose gel electrophoresis. Using high-capacity cDNA reverse-transcription kit (ThermoFisher Scientific), 1µL of total RNA was reverse transcribed. cDNA was diluted into 1:3 ratio for IL-6 gene. Cq values of each gene was obtained using 2 µL of diluted cDNA in a total 20µL TaqMan Fast Advanced Reaction Mix (Applied Biosystems) on QuantStudio™ 3 system. Each reaction was run in duplicate. Predesigned primers for IL-6 (Mm00446190_m1), was purchased from Applied Biosystems. Data were analyzed using QuantStudio™ analysis software. Ct value of target gene was normalized with internal control gene, and fold change expression was calculated with 2^−ddCq^ method.

### Quantification of LPS/endotoxin

LPS (endotoxin lipopolysaccharide) quantification was carried out per manufacturer’s instructions (Thermoscientific, Pierce™ Chromogenic Endotoxin Quant Kit, Catalog number A39553, Waltham, MA). All reagents were equilibrated to room temperature before use. Great care was taken to use materials that are endotoxin free. A standard of known endotoxin concentrations (0.100–0.010 EU/mL) was run in parallel with samples. Plasma samples were diluted at 1:50 in endotoxin free water supplied by the kit, and then heat shocked at 70 °C for 15 min before use. For the duration of this test the 96 wells plate was kept at 37 °C (Corning LSE Digital Dry Bath). Following colorimetric change wells were read at a wavelength of 405 nm using Bio-Rad xMark™ Microplate Absorbance Spectrophotometer.

### Statistical analysis

Quantitative data were expressed as mean ± standard deviation (SD) unless otherwise stated, and statistical differences between more than two groups were determined by analysis of variance (ANOVA) followed by multiple comparisons using Tukey's multiple comparisons test. For ELISA, a comparison between two samples was performed by unpaired *t* test. GraphPad Prism version 9.1.0 for Windows (GraphPad Software, Inc., San Diego, CA) was used to perform the statistical analysis. Differences with *p* < 0.05 were considered significant (*p < 0.05, **p < 0.01, ***p < 0.001, ****p < 0.0001).

## Results

We first established a mouse model to evaluate UTI-induced neurological effects using a well-characterized model of UTI that has previously been used by other groups to study UTI-related pathology [9]. We used only female mice to reflect the population at highest risk for developing UTI [8] and a uropathogenic *E. coli* model, which is the most predominant infectious pathogen in UTI [10, 11].

**Fig. 1:**
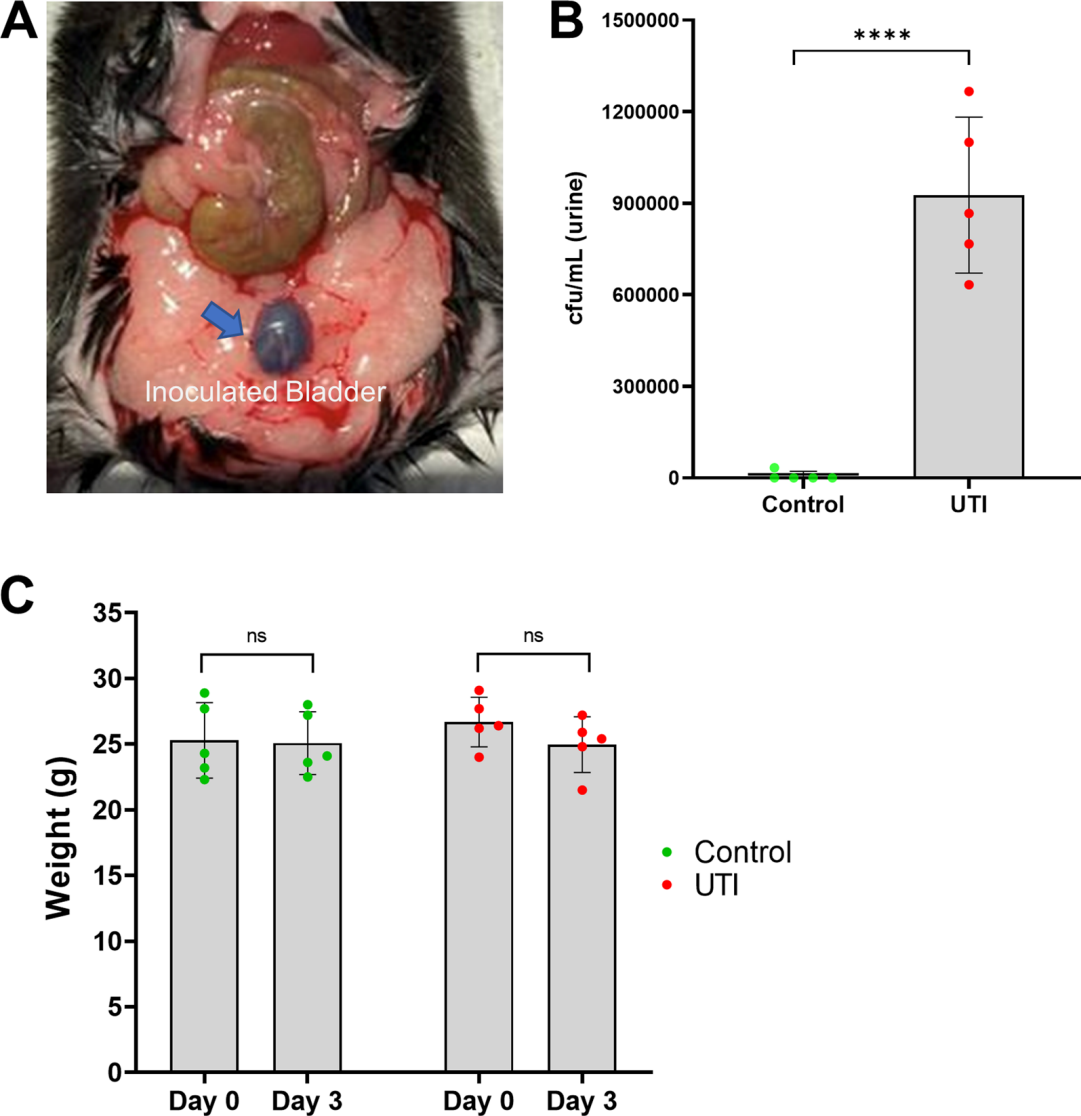
Transurethral inoculation of *E. coli* causes UTI. **a** Representative image of mouse bladder (blue arrow) following successful transurethral inoculation of Evans Blue dye. **b** 72 h post-infection, urine was aseptically collected to enumerate bacterial burden as colony forming unit (cfu) which showed significantly increased bacterial count in the UTI group compared to the control group (*n* = 5/group). **c** Mean weights of mice on the day of inoculation (day 0) and 72 h post-inoculation (day 3) indicate no significant weight loss due to UTI. Quantitative data are expressed in mean ± SD, ****p < 0.0001

Validation of the transurethral procedure of inoculation into the bladder was confirmed by Evans Blue dye (Fig. 1a). No bacteria were recovered from the tissues (bladder and kidneys) or urine of the mice inoculated with sterile PBS (control), while there was significantly increased formation of bacterial colonies in the mice who were transurethrally inoculated with 10^8^ cfu/mL uropathogenic *E. coli* into the bladder (Fig. 1b). The organism persisted in the urinary bladder, kidneys, and urine samples after 72 h with a mean colony count of approximately 10^6^. After UTI induction, the physiological status of each mouse was monitored carefully—no signs of systemic illness, such as weight loss, were observed in any of the mice (Fig. 1c). Plasma level of endotoxin was quantified using chromogenic endotoxin quantification kit and found to be significantly elevated in the UTI mice compared to non-UTI controls (Additional File 1: Fig. S1).

**Fig. 2:**
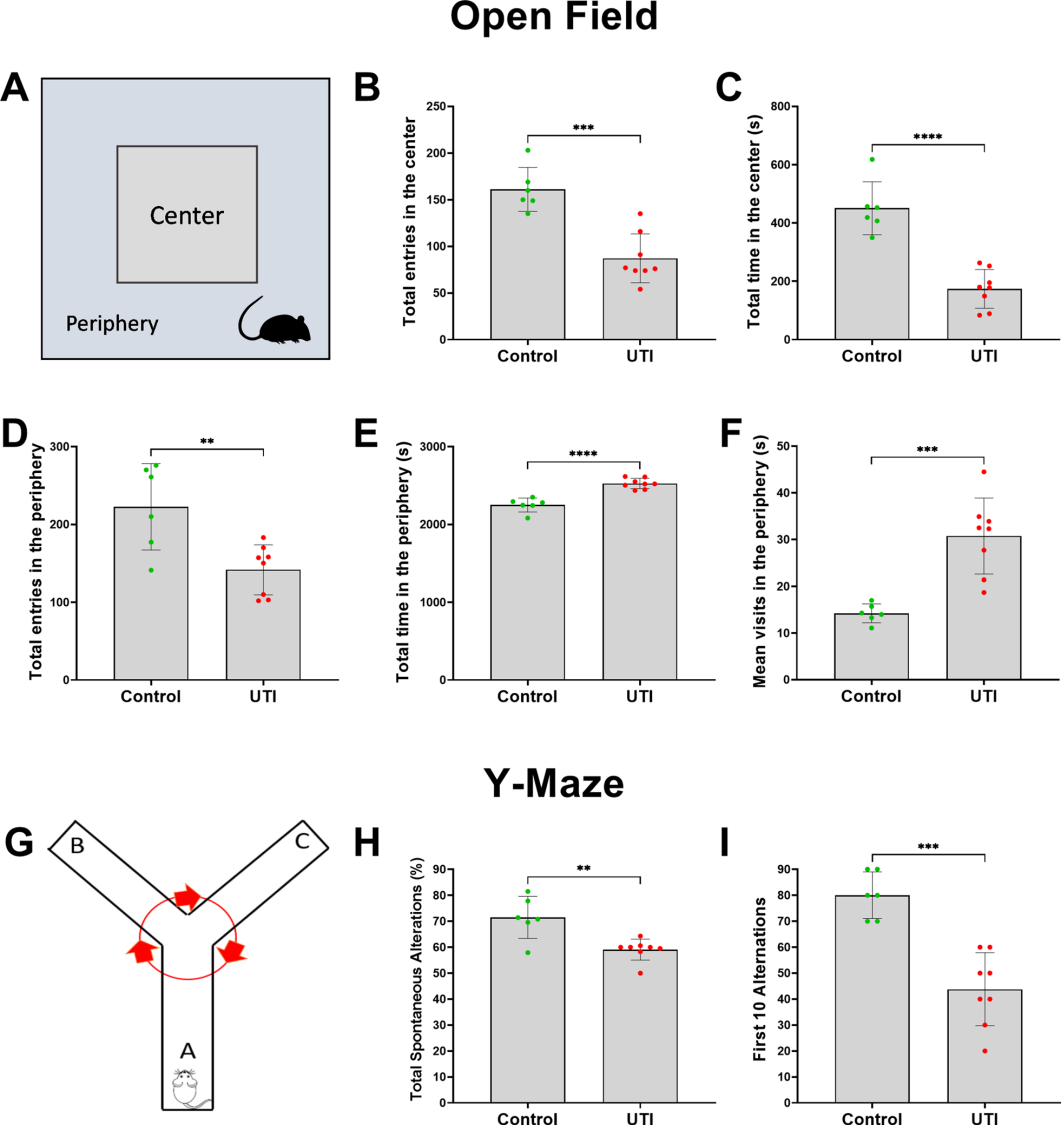
UTI induces frontal and hippocampal functional impairments in the Open Field and Y-Maze tests. **a** Schematic of the apparatus and assessment of behavioral function in center and peripheral zones of the Open Field. **b, c** In the open field behavioral test, UTI mice entered and spent significantly less time in the center zone compared to the control group. **d**–**f** In the Open Field behavioral test, although UTI mice had fewer total entries into the peripheral zone, they spent more total time and had more mean visit times in the periphery than control group. **g** Schema of the Y-maze apparatus and behavioral assessments. **h, i** In the Y-maze test, total spontaneous alternations in 5 min and the first ten alternations were markedly reduced in the UTI group in comparison to the control group. Each dot represents one animal (*n* = 6 in control, *n* = 8 in UTI). Behavioral tests were performed 48 h after inoculation. Quantitative data are expressed in mean ± SD. *p < 0.05, **p < 0.01, ***p < 0.001, ****p < 0.0001

To assess the functional neurological effects of UTI, we performed the Open Field and Y-maze tests. Compared to non-UTI controls, mice with UTI spent significantly more time in the periphery of the maze, in proximity to the walls, and less time in the center area, indicating increased thigmotaxis or anxiety (Figs. 2a–f). There were significantly fewer entries to both center and peripheral zones in the mice with UTI compared to non-UTI controls (Fig. 2b, d). Despite this, the total time spent and mean visits to the peripheral zone were significantly increased in the UTI group compared to the non-UTI control group (Fig. 2e, f). The Y-Maze spontaneous alternation is widely used as an assessment of short-term memory or inattention [12]. Compared to non-UTI controls, mice with UTI exhibited significantly fewer spontaneous alternations in the first 10 alternations that persisted for the entire 5-min duration of the Y-Maze test (Fig. 2g–i).

**Fig. 3:**
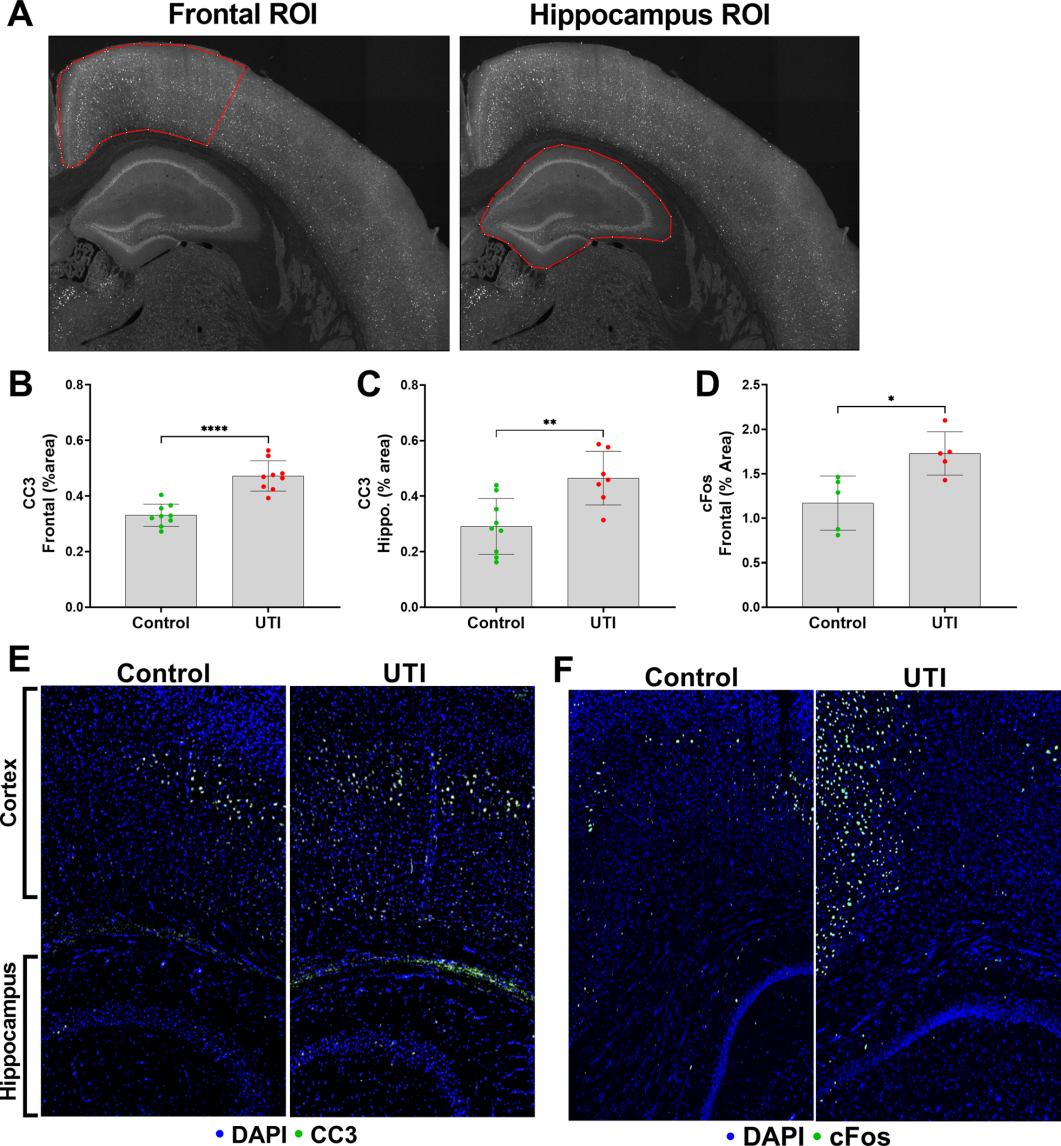
UTI induces neuronal changes to the frontal cortex and hippocampus. **a** Representative regions of interest (ROIs) of measured brain regions, i.e., frontal cortex, and hippocampus. **b**, **c** Quantification of cleaved caspase-3 (CC3) in the frontal cortex and the hippocampus show significantly increased CC3 in the UTI group compared to the control group (*n* = 7–9). **d** Quantitative data show significant increase in c-Fos in frontal cortex area in the UTI group compared to the control group (*n* = 5–9). **e**, **f** Representative images of CC3 and c-Fos (both shown in green) in both the frontal cortex and hippocampus show increases in both markers in UTI mice compared to controls. Quantitative data are expressed in mean ± SD. *p < 0.05, **p < 0.01, ***p < 0.001, ****p < 0.0001

**Fig. 4:**
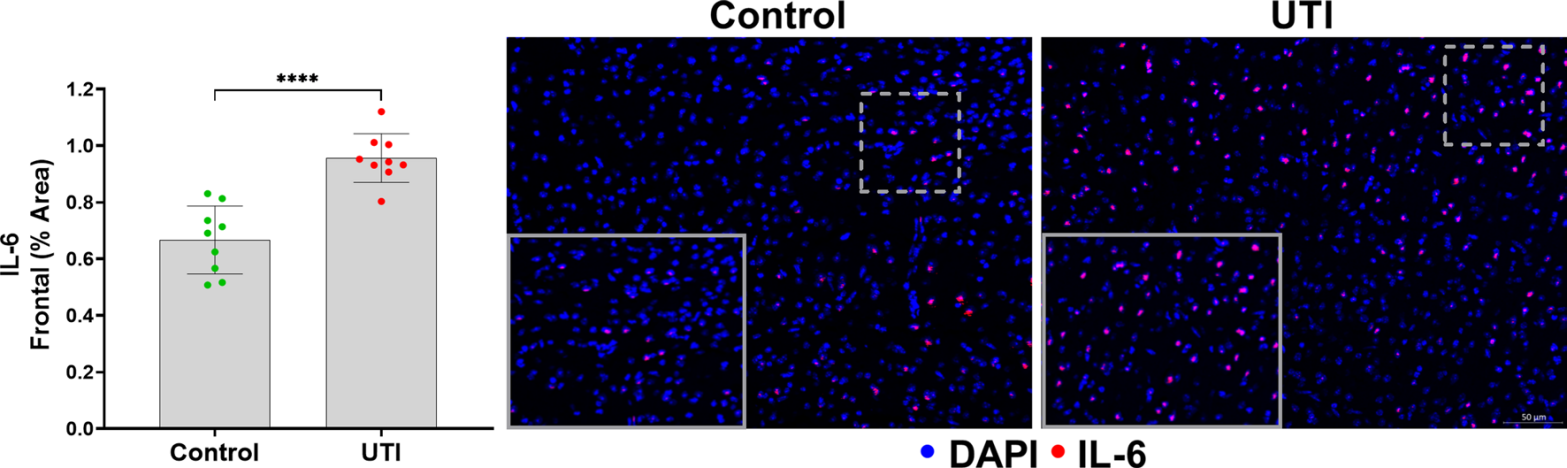
UTI increases IL-6 within the frontal cortex. Quantitative data show significant increase in frontal IL-6 in UTI mice compared to non-UTI mice. Representative image demonstrates increases in IL-6 within the frontal cortex (*n* = 9). Areas delineated by dashed lines are magnified and shown in the lower left corner of the image. Quantitative data are expressed in mean ± SD, ****p < 0.0001

We then quantified neuronal cleaved caspase-3 (CC3) in the frontal cortex and hippocampus, as previously characterized by our laboratory [7]. An overview of the representative analyzed regions of interest of our UTI-induced neuronal changes model are shown in Fig. 3a**.** The caspases are a family of protease enzymes that when activated, such as to CC3, can trigger apoptotic pathways that execute neuronal death [13, 14]; however, CC3 has also been described as a regulatory molecule that contributes to neurogenesis and synaptic activity [15, 16]. CC3 and c-Fos, a transcription factor that is upregulated with neuronal activity, expressions were significantly increased in the frontal cortex and hippocampus of the UTI group compared to the non-UTI control group (*p* < 0.0001) (Fig. 3b–f). To examine a role for the delirium-relevant inflammatory marker, IL-6, we first characterized the time course of IL-6 release in the systemic circulation over 72 h of UTI. At baseline, IL-6 was < 10 pg/mL, which significantly increased over 36 h of UTI (*p* < 0.01), followed by a partial decrease at 72 h that was still significantly increased compared to baseline (*p* < 0.05) (Additional File 1: Fig. S2). To characterize the brain changes associated with UTI, we measured the cortical expression of IL-6 [17, 18], which was significantly increased in the UTI group compared to the non-UTI control group (Fig. 4).

**Fig. 5:**
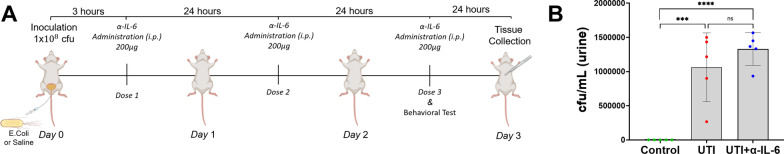
Inoculation and anti-IL-6 treatment regimen. **a** Schematic of experimental design and treatment schedule for anti-IL-6 antibody. **b** After 72 h of infection, there was no significant change in bacterial count in the UTI group compared to the anti-IL-6 antibody treated group

**Fig. 6:**
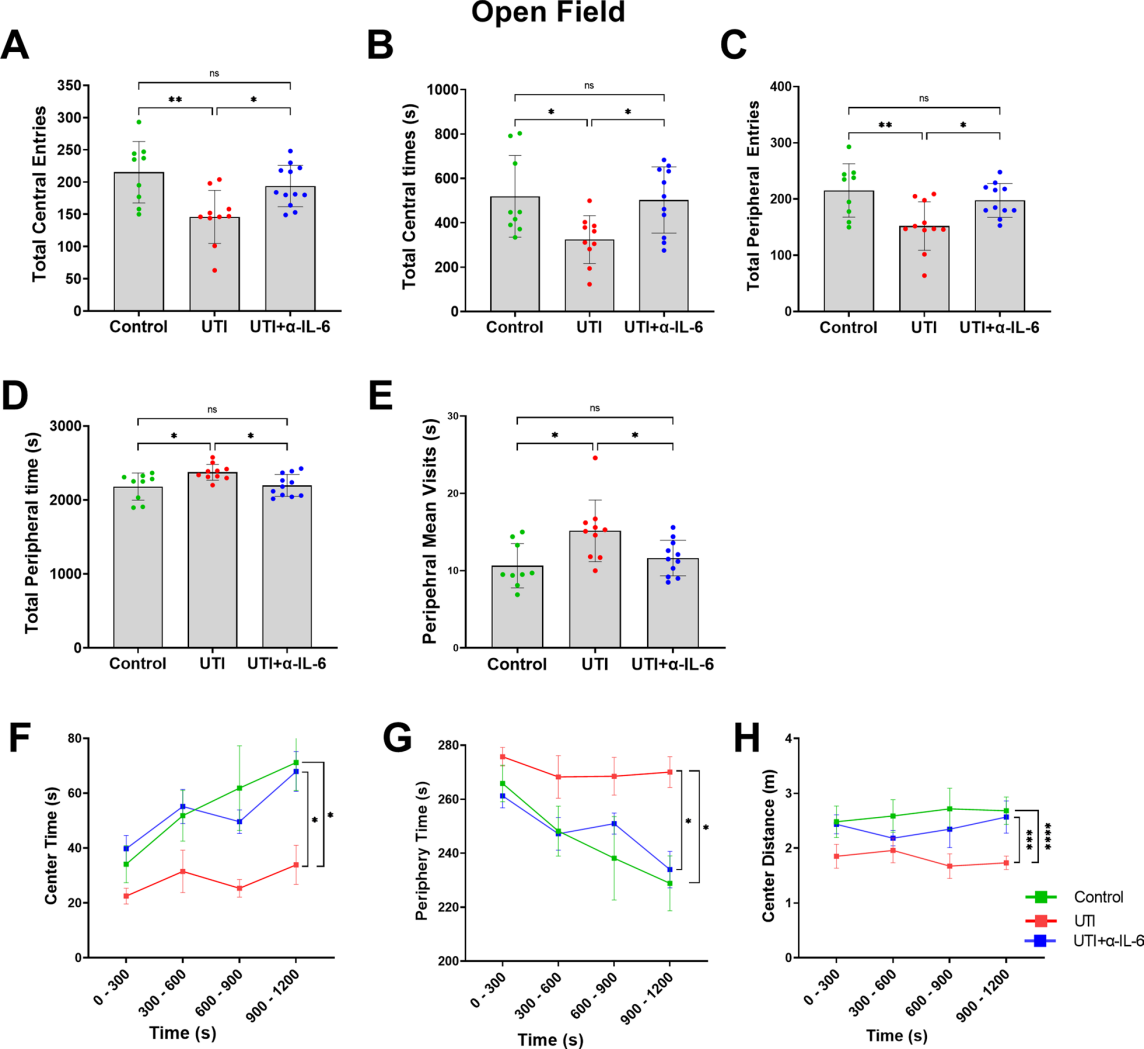
Systemic IL-6 inhibition significantly improves UTI-induced impairments in the Open Field test. **a**, **b** In the open field behavioral test, UTI mice entered (ANOVA, *F* = 7.64, dF = 30, *p* = 0.0023) and significantly less time (ANOVA, *F* = 4.942, dF = 29, *p* = 0.0148) in the center zone compared to the control or UTI + α-IL-6 groups. **c**–**e** In the Open Field behavioral test, although UTI mice had fewer total entries into the peripheral zone (ANOVA, *F* = 6.728, dF = 30, *p* = 0.0041), they spent more total time (ANOVA, *F* = 9.889, dF = 29, *p* = 0.0006) and had more mean visit times in the periphery than control or UTI + α-IL-6 groups (ANOVA, *F* = 5.671, dF = 29, *p* = 0.0088). **f–h** Line graphs for mean time for every 5 min segment in the center and peripheral zones show that UTI mice spent significantly less time in the center and more time in the periphery compared to the control group, indicating more psychomotor agitation in the UTI mice. In contrast, treatment with α-IL-6 antibody was able to reduce the time spent in the periphery, suggesting less anxiety. Behavioral tests were performed 48 h after inoculation. Quantitative data are expressed in mean ± SD. *p < 0.05, **p < 0.01, ***p < 0.001, ****p < 0.0001

**Fig. 7:**
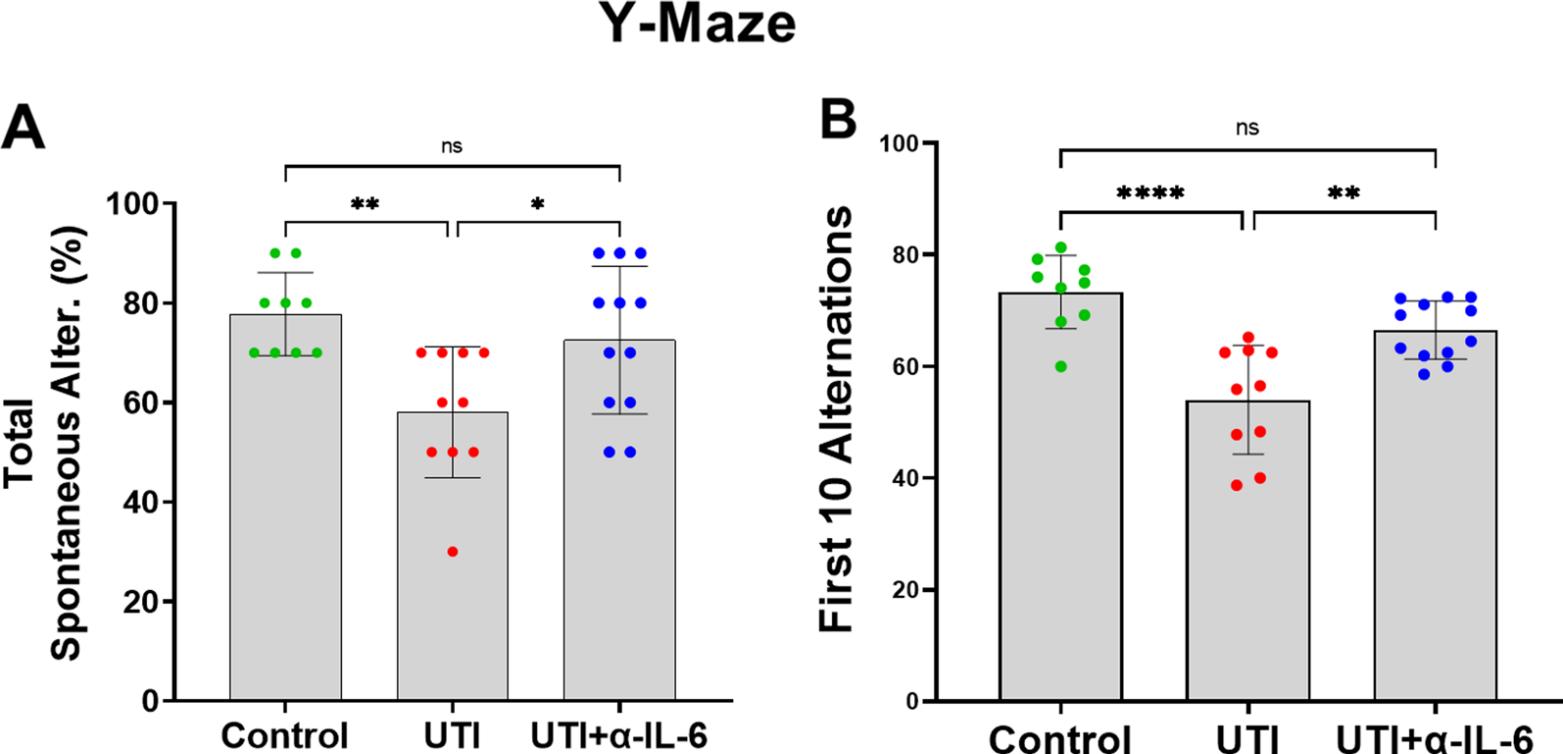
Systemic IL-6 inhibition significantly improves UTI-induced impairments in the Y-maze test. **a**, **b** In the Y-maze test, total spontaneous alternations in 5 min (ANOVA, *F* = 6.310, dF = 30, *p* = 0.0085) and the first ten alternations (ANOVA, F = 17.18, dF = 30, *p* < 0.0001) were markedly reduced in the UTI group in comparison to the control or UTI + α-IL-6 groups. Each dot represents one animal (*n* = 9 in control, *n* = 10 in UTI, *n* = 12 in UTI + α-IL-6). Behavioral tests were performed 48 h after inoculation. Quantitative data are expressed in mean ± SD. *p < 0.05, **p < 0.01, ****p < 0.0001

In a separate cohort of animals, we then examined the role of systemic IL-6 in mediating delirium-like functional and structural phenotypes using three experimental groups: (1) non-UTI control, (2) UTI, and 3) UTI + anti-IL-6 antibody (Fig. 5a). There was no significant difference in the urinary bacterial counts between the UTI mice treated with saline (UTI group) and the UTI mice treated with anti-IL-6 antibody (UTI + α-IL-6 Ab group) (Fig. 5b). UTI mice treated with anti-IL-6 antibody spent significantly more time in the center compared to the periphery of the maze compared to mice with UTI, similar to what was measured in control non-UTI mice (Fig. 6a–h). In addition, there were significantly fewer entries to both center and peripheral zones in the UTI group compared to control mice or those treated with anti-IL-6 antibody (Fig. 6a, c), while the total time spent and mean visits to the peripheral zone was markedly increased in the UTI group compared to other groups (Fig. 6d, e). Analyses using 5-min time segments showed that UTI mice treated with anti-IL-6 antibody or control non-UTI mice demonstrated a more consistent increase in preference for the center zone of the maze throughout the duration of the analysis, while UTI mice demonstrated sustained preference for the periphery (Fig. 6f–h). In the Y-Maze test, compared to UTI mice, mice treated with anti-IL-6 antibody or control non-UTI mice exhibited significantly greater spontaneous alternations in the first 10 alternations that lasted for the entire 5-min duration of the Y-Maze test (Fig. 7a, b).

To evaluate any independent effects of anti-IL-6 antibody on CC3 and behavioral function, we administered anti-IL-6 antibody or saline to non-UTI wild-type mice (*n* = 6/group) and found no difference in frontal/hippocampal CC3 expression or behavioral function (Additional file 1: Fig. S3A–M).

To investigate any potential confounding effects of UTI on general level of activity, we evaluated several distinct locomotor parameters in the Open Field and Y-maze. There were no significant differences in total distance traveled, average speed, maximum speed, total mobile time, or total immobile time between the groups (Additional file 1: Fig. S4A–E) in the Open Field test, while in the Y-maze, there were no significant differences in the number of arm entries (Additional file 1: Fig. S4F), and no significant correlation between spontaneous alternation and the number of arm entries across all three experimental groups (Additional file 1: Fig. S4G). Overall, these data suggest that differences in spontaneous locomotor activity did not influence measurements of behavioral function and that any differences in functional testing can be attributed to cognitive deficits rather than a limitation of activity [19].

**Fig. 8:**
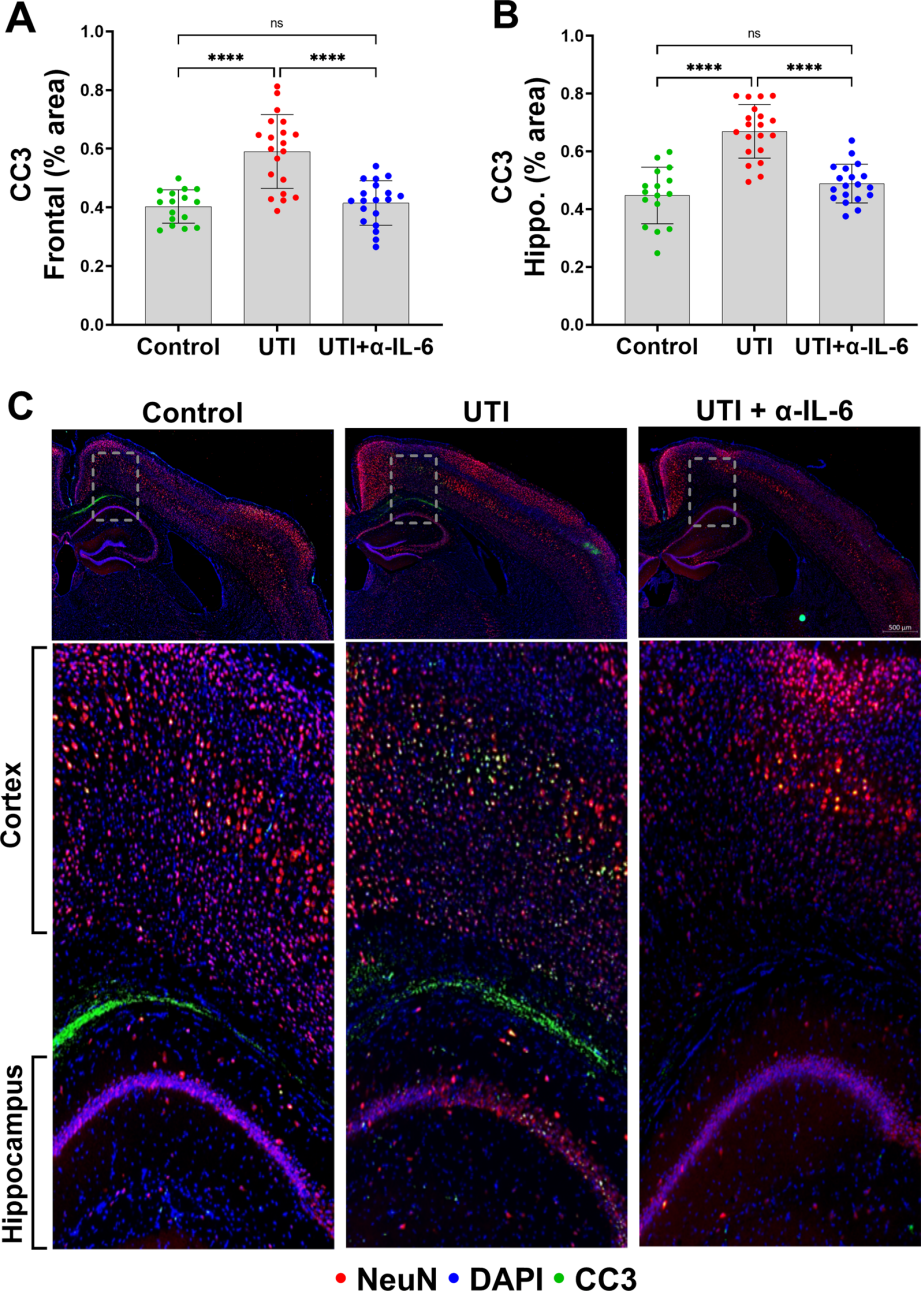
Systemic IL-6 inhibition fully reverses UTI-induced neuronal changes to the frontal cortex and hippocampus. **a**, **b** Quantification of cleaved caspase-3 (CC3) in the frontal cortex (ANOVA, *F* = 24.01, dF = 54, *p* < 0.0001) and the hippocampus (ANOVA, *F* = 34.92, dF = 54, *p* < 0.0001) showed significant reduction of CC3 expression in the anti-IL-6 antibody treated group compared to the UTI group. Each dot represents one animal (*n* = 16 in control, *n* = 20 in UTI, *n* = 19 in UTI + α-IL-6). **c** Representative sections stained for CC3 (positive signal displayed in green) with both the neuronal marker, NeuN (red), and DAPI nuclear stain (blue). Magnified regions, indicated by dashed lines, of an area including both the frontal neocortex and hippocampus from the micrograph directly above reveal CC3 expression within neurons. Quantitative data are expressed in mean ± SD, ***p < 0.001, ****p < 0.0001

**Fig. 9:**
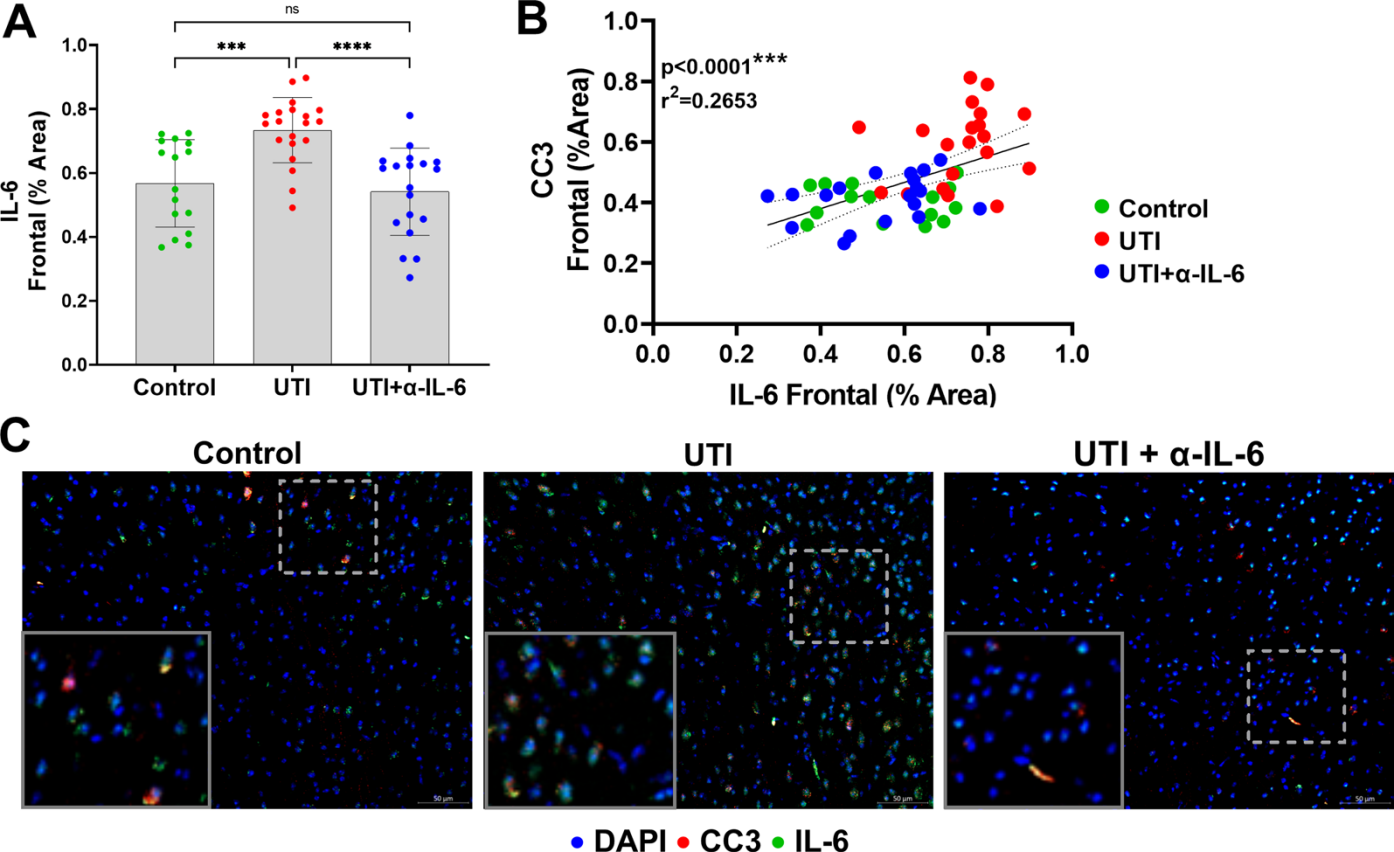
Systemic IL-6 inhibition significantly reduces UTI-induced upregulation of frontal IL-6. **a** IL-6 is significantly increased in the frontal cortices of the UTI group compared to the control and UTI + α-IL-6 groups. **b** Correlational analysis demonstrate significant positive relationships between frontal cortical CC3 with IL-6. **c** Representative micrographs of frontal cortical neurons stained for IL-6 (displayed in green) and CC3 (displayed in red). Magnified areas of individual neurons and glial cells are inset. Note that CC3-positive neurons also co-stain for IL-6 in UTI brains. Each dot represents one animal (*n* = 16 in control, *n* = 20 in UTI, *n* = 19 in UTI + α-IL-6). Quantitative data are expressed in mean ± SD. ***p < 0.001, ****p < 0.0001

Immunohistochemical analysis showed that treatment of the UTI group with anti-IL-6 antibody fully reversed the frontal and hippocampal CC3 changes compared to the UTI group (*p* < 0.0001) (Figs. 8a, b). Representative micrographs stained for CC3 and neurons (NeuN) are shown in Fig. 8c. There was a significant increase in frontal IL-6 expression in mice with UTI compared to control animals (*p* = 0.0007) (Fig. 9a). Treatment of the UTI group with anti-IL-6 antibody resulted in a significant reduction of frontal IL-6 (*p* < 0.0001), which were confirmed with ELISA (Additional file 1: Fig. S5A). Of note, IL-6 expression was mostly localized within neurons, while IL-1β localized to the intercellular space of the brain parenchyma. IL-6 was significantly and positively correlated with CC3 (*r*^2^ = 0.2653/*p* < 0.0001) in the frontal cortex, (Fig. 9b). There was an apparent colocalization between IL-6 and CC3-positive frontal cortical neurons (Fig. 9c). Quantification of cortical IL-6 mRNA demonstrated no significant differences across the three experimental groups, suggesting systemic derivation of the measured cortical IL-6 rather than from synthesis within the brain, which is consistent with amelioration of neuronal changes following systemic IL-6 inhibition (Additional file 1: Fig. S5B).

**Fig. 10:**
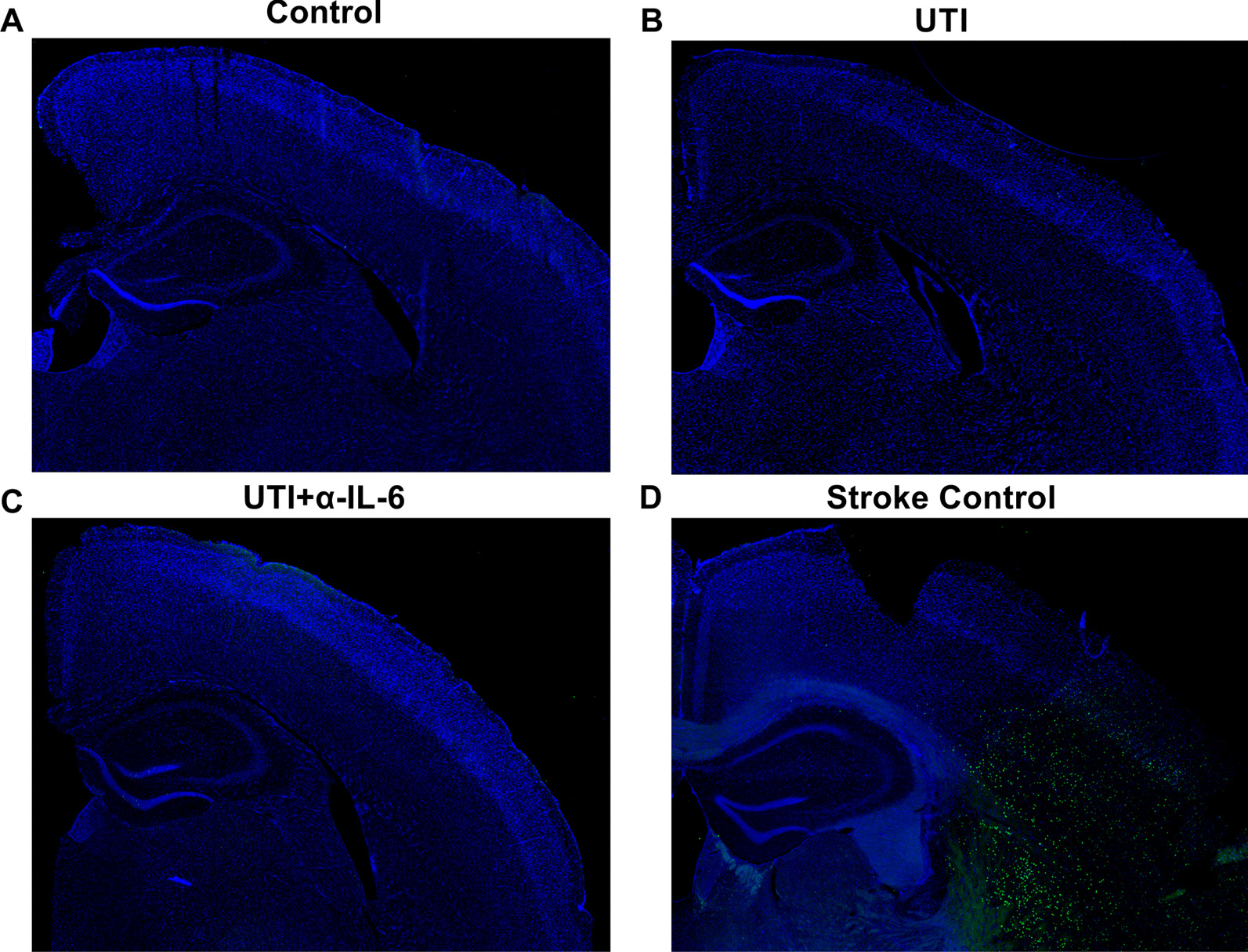
There is no neuronal cell death in mice following UTI. **a**–**d** TUNEL staining (green) on brain sections of UTI mice compared to a brain section from an ischemic stroke mouse model shows high levels of cell death within the infarct area, but no cellular death in the frontal cortex or hippocampus of UTI mice

There was no evidence of cell death indicated by the absence of Terminal deoxynucleotidyl transferase dUTP nick end labeling (TUNEL) staining for detecting apoptotic DNA fragmentation in the UTI mice compared to a mouse brain section from an acute ischemic stroke model (TUNEL-positive control) (Fig. 10a–d).

**Fig. 11:**
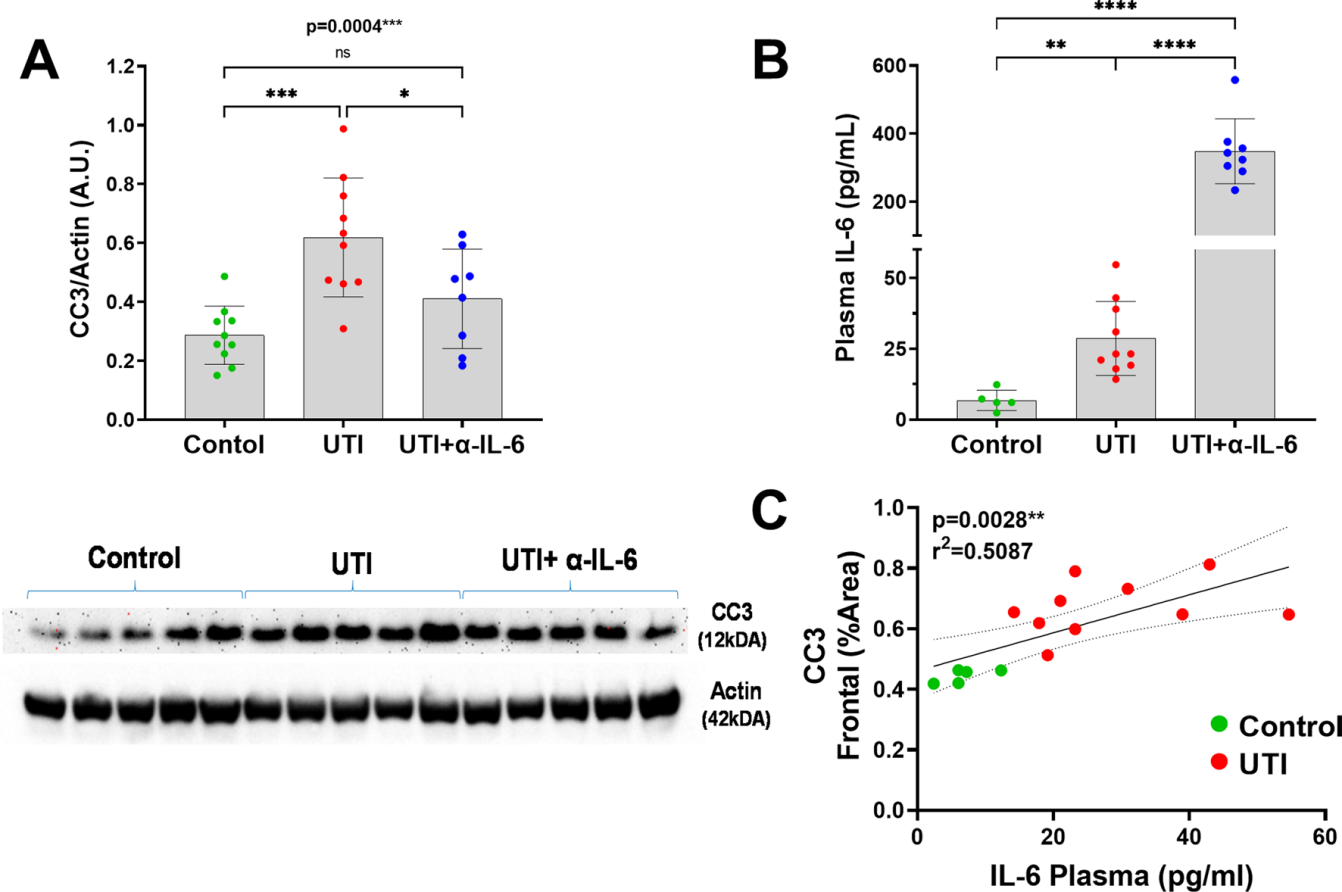
Western blot confirms immunohistological CC3 findings and plasma IL-6 levels positively correlate with cortical CC3. **a** Quantification (upper panel) and representative western blot images (lower panel) of cortical CC3 levels shows significant increases in UTI mice treated with saline (*p* < 0.001) (ANOVA, F = 85.28, dF = 22, *p* < 0.0001) and significant reduction of cortical CC3 in UTI mice treated with the anti-IL-6 antibody compared to the control group (*p* < 0.05) (ANOVA, *F* = 10.75, dF = 27, *p* = 0.0004). Each dot represents one animal (*n* = 10 in control, *n* = 10 in UTI, *n* = 8 in UTI + α-IL-6). **b** Plasma IL-6 levels measured via ELISA shows UTI and UTI + α-IL-6 group had significantly increased plasma IL-6 compared to the control group (*n* = 5 in control, *n* = 10 in UTI, *n* = 8 in UTI + α-IL-6). Quantitative data are expressed in mean ± SD. **c** Positive significant correlation between plasma IL-6 and frontal CC3 for UTI mice and controls. *p < 0.05, **p < 0.01, ***p < 0.001, ****p < 0.0001

Western blot confirmed the immunohistochemistry data demonstrating significantly increased CC3 concentration in the UTI group compared to control mice or UTI mice treated with anti-IL-6 antibody (ANOVA *p* = 0.0004) (Fig. 11a). Plasma IL-6 concentrations were significantly elevated in UTI mice compared to controls, while plasma IL-6 concentrations were most significantly elevated in UTI mice who received anti-IL-6 antibody, which is most likely due to impaired clearance of IL-6/anti-IL-6 complex (Fig. 11b) [20]. After excluding the anti-IL-6 antibody treated group, there was a strong positive and significant correlation between plasma IL-6 and CC3 (*r*^2^ = 0.5087/*p* = 0.0028) (Fig. 11c).

## Conclusions

This study provides evidence demonstrating a pathological role for the IL-6 signaling pathway in mediating functional and structural delirium-like phenotypes in an animal model of UTI. These findings suggest that pharmacological modulation of systemic IL-6 may mitigate delirium-like functional and structural phenotypes after UTI and serve as pre-clinical justification for clinical investigations using IL-6 inhibition to treat UTI-induced delirium.

There are currently few effective and evidence-based treatments for delirium. Although ubiquitously used, anti-psychotic medications, such as haloperidol, have been demonstrated to confer no significant benefit to clinical outcomes related to delirium compared to placebo [21, 22]. Current clinical paradigms to mitigate delirium rely on treatment of the precipitating cause, reduction or elimination of sedatives or other deliriogenic medications, liberation from the mechanical ventilator, and optimization of sleep and environmental conditions [23–26]. While these protocols have a modest effect on reducing delirium, they are logistically challenging to consistently implement and resource intensive, resulting in poor adherence. The findings of this study suggest that pharmacological modulation of the IL-6 pathway may offer an opportunity to mitigate delirium, for which there are several potential advantages. First, delirium is well-known to independently contribute to high mortality and prolonged length of stay [27], thus, it is possible that amelioration of delirium will improve these important outcomes. Furthermore, it is also known that UTI-induced delirium may represent a crucial tipping point, where sub-clinical or mild cognitive impairment converts to long-term dementia and that, conversely, patients with dementia are particularly susceptible to developing both UTI as well as UTI-induced delirium [28–34]. It is, thus, important to determine to what extent these conditions contribute bidirectionally to the pathogenesis of the other, and whether treatment paradigms directed at reducing rates of UTI-induced delirium can improve long-term cognitive function.

Our data provide optimism that the functional signs of delirium may precede irreversible neuronal death, as evidenced by no TUNEL-positive staining to suggest apoptotic neuronal death. These findings may further indicate the existence of a window of opportunity within which implementation of timely interventions may prevent long-term or permanent brain injury and is consistent with the clinical picture of delirium as a manifestation of potentially reversible cerebral stress [7].

There are notable limitations of this study that warrant consideration. First, we used relatively young mice who do not represent the older population that is at highest risk for developing UTI-induced delirium; however, we expect older mice to demonstrate disproportionately severe delirium-like phenotypes given that advanced age is a strong risk factor for delirium. Second, we did not assess the effect of antibiotic treatments; however, it is also a clinical reality that the diagnosis of UTI and/or initiation of antibiotics is often delayed and that clinical symptoms of delirium often start before and persist long after initiation of antibiotics. Furthermore, antibiotic resistant strains of UTI are increasingly prevalent and may not sufficiently neutralize the UTI. We also did not vary the time of exposure to UTI, which may affect the response to systemic IL-6 inhibition. Finally, we do not know if IL-6 mediates neuronal changes directly or indirectly through intermediate cell types, such as glial cells. Since neurons do not express the IL-6 receptor, we speculate that the IL-6/IL-6R trans-signaling pathway via gp130 on neurons may be critical in mediating cognition-relevant neuronal damage, similar to what has been described in a mouse model of dementia [35, 36]. Overall, it is likely that IL-6 emerges from macrophages and epithelial cells as an acute phase response to UTI-induced endotoxin release in the blood [37, 38] and that the anti-IL-6 neutralization occurs peripherally, rather than in the brain. The relatively large size of the anti-IL-6 antibody (~ 150kDA) makes it unlikely to cross the blood–brain barrier to act directly on the brain.

In summary, this study provides first evidence that systemic IL-6 mediates delirium-like phenotypes in an animal model of UTI. These findings justify future investigations to test the efficacy of modulating the IL-6 signaling pathway to ameliorate UTI-induced delirium.

## Supplementary Information

**Additional file 1: Figure S1. **The plasma level of endotoxin was significantlyelevated in the UTI mice compared to non-UTI controls. The endotoxin level was quantifiedusing a chromogenic endotoxin quantification kit. Quantitative data areexpressed in mean ± SD. ****p < 0.0001. **Figure S2. **UTI causes an increase in plasma IL-6 levels by 36 hourspost-inoculation that is sustained at 72 hours. Blood was collected at baseline, 36-, and 72-hours post-inoculation andplasma IL-6 levels were quantified via ELISA. The data show that there is anapproximate, and significant 8-fold increase in plasma IL-6 acutely followingtransurethral inoculation that persists at 72 hours, which is the final timepoint for all other cohorts. Quantitative data are expressed in mean ± SD. *p< 0.05, **p < 0.01. **Figure S3**. α-IL-6 treatment to non-UTI micecauses no change in either brain histology or behavior. Non-inoculated,wild-type mice were given the same α-IL6 antibody treatments as the UTI cohort,i.e., once daily doses for 3 days. A-B: IHC analysis of CC3 levels within thefrontal cortex and hippocampus showed no difference in animals treated with theantibody compared to those treated with saline (vehicle). C-M: The miceunderwent behavioral testing (i.e., open field and Y-Maze) and treated miceshowed no difference in behavior compared to controls. Quantitative data areexpressed in mean ± SD. **Figure S4**. There is no significant difference in overalllocomotor activity in UTI compared to control or UTI+α-IL-6 groups. A-E: Quantitative dataof open field behavioral test show no significant difference in total distancetraveled, average speed, maximum speed, total mobile time, or total immobiletime between the groups. F-G: Quantitative dataof Y-maze behavioral test showing no significant change in the total number ofentries in different arms between the experimental groups. Each dot representsone animal (n=9 in control, n=10 in UTI, n=12 in UTI+α-IL-6). **Figure S5**. A: Quantitativedata via ELISA corroborate immunohistochemistry findings by showing significantmitigation of frontal IL-6 in UTI mice treated with systemic anti-IL-6antibody compared to UTI mice. B: Quantitative data via RT-PCR show nosignificant differences in cortical IL-6 mRNA across the three experimentalgroups. Quantitative data are expressed in mean ± SD. *p < 0.05, ****p <0.0001.

## Data Availability

The data sets used and/or analyzed during the current study are available from the corresponding author on reasonable request.

## Abbreviations

UTI: Urinary tract infection
IL: Interleukin
CC3: Cleaved caspase-3
TUNEL: Terminal deoxynucleotidyl transferase dUTP nick end labeling
*E. coli*: *Escherichia coli*
ANOVA: Analysis of variance
ROI: Regions of interest
