# Supplementary material for: Interleukin-6 mediates delirium-like phenotypes in a murine model of urinary tract infection
Source: J Neuroinflammation. 2021 Oct 28;18:247. doi: 10.1186/s12974-021-02304-x (PMC8554965; doi:10.1186/s12974-021-02304-x)
Supplement: Supplementary file 1 — Additional file 1: Figure S1. The plasma level of endotoxin was significantlyelevated in the UTI mice compared to non-UTI controls. The endotoxin level was quantifiedusing a chromogenic endotoxin quantification kit. Quantitative data areexpressed in mean ± SD. ****p < 0.0001. Figure S2. UTI causes an increase in plasma IL-6 levels by 36 hourspost-inoculation that is sustained at 72 hours. Blood was collected at baseline, 36-, and 72-hours post-inoculation andplasma IL-6 levels were quantified via ELISA. The data show that there is anapproximate, and significant 8-fold increase in plasma IL-6 acutely followingtransurethral inoculation that persists at 72 hours, which is the final timepoint for all other cohorts. Quantitative data are expressed in mean ± SD. *p< 0.05, **p < 0.01. Figure S3. α-IL-6 treatment to non-UTI micecauses no change in either brain histology or behavior. Non-inoculated,wild-type mice were given the same α-IL6 antibody treatments as the UTI cohort,i.e., once daily doses for 3 days. A-B: IHC analysis of CC3 levels within thefrontal cortex and hippocampus showed no difference in animals treated with theantibody compared to those treated with saline (vehicle). C-M: The miceunderwent behavioral testing (i.e., open field and Y-Maze) and treated miceshowed no difference in behavior compared to controls. Quantitative data areexpressed in mean ± SD. Figure S4. There is no significant difference in overalllocomotor activity in UTI compared to control or UTI+α-IL-6 groups. A-E: Quantitative dataof open field behavioral test show no significant difference in total distancetraveled, average speed, maximum speed, total mobile time, or total immobiletime between the groups. F-G: Quantitative dataof Y-maze behavioral test showing no significant change in the total number ofentries in different arms between the experimental groups. Each dot representsone animal (n=9 in control, n=10 in UTI, n=12 in UTI+α-IL-6). Figure S5. A: Quantitativedata via ELISA corroborat [file 12974_2021_2304_MOESM1_ESM.docx]

**Interleukin-6 Mediates Delirium-Like Phenotypes in a Murine Model of Urinary Tract Infection**

Mohammad Harun Rashid^1^, Nicklaus A. Sparrow^1^, Faizan Anwar^1^, Gena Guidry^1^, Ambart E. Covarrubias^2^, Haoming Pang^1^, Chandrakumar Bogguri^3^, S. Ananth Karumanchi^2^, Shouri Lahiri^4^**^*^**

**Online Data Supplement**

**Supplemental Figures**

**Additional File, Figure S1**


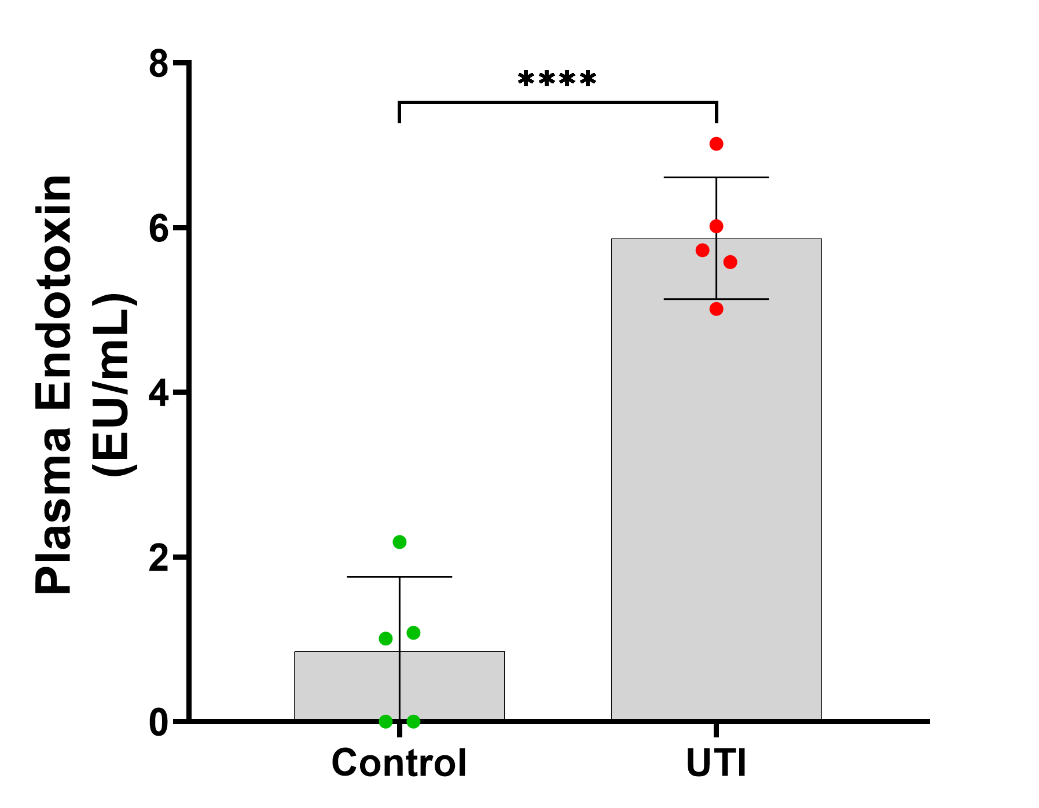


The plasma level of endotoxin was significantly elevated in the UTI mice compared to non-UTI controls. The endotoxin level was quantified using a chromogenic endotoxin quantification kit. Quantitative data are expressed in mean ± SD. ****p < 0.0001.

**
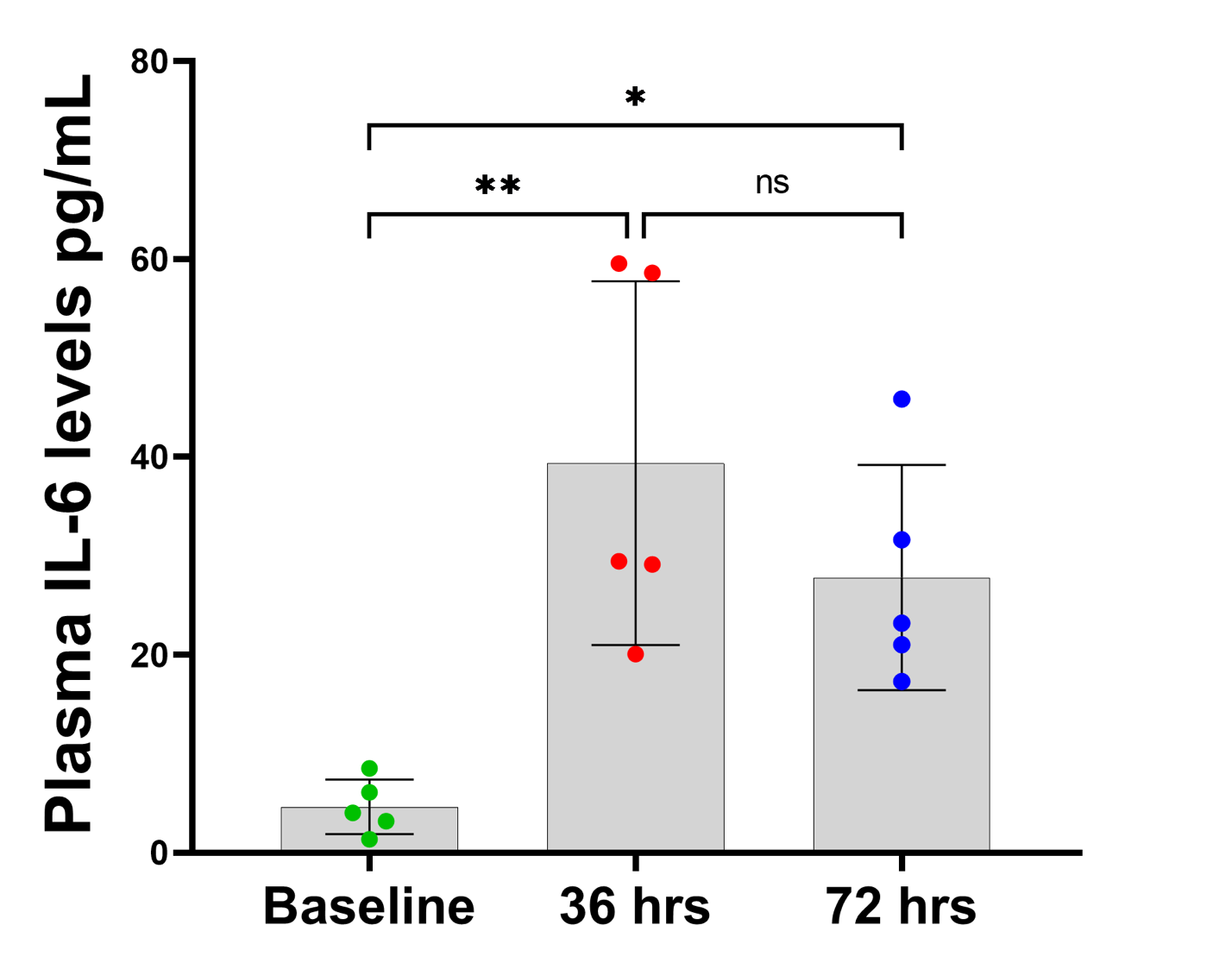
Additional File, Figure S2**

**UTI causes an increase in plasma IL-6 levels by 36 hours post-inoculation that is sustained at 72 hours.** Blood was collected at baseline, 36-, and 72-hours post-inoculation and plasma IL-6 levels were quantified via ELISA. The data show that there is an approximate, and significant 8-fold increase in plasma IL-6 acutely following transurethral inoculation that persists at 72 hours, which is the final time point for all other cohorts. Quantitative data are expressed in mean ± SD. *p < 0.05, **p < 0.01.

**Additional File, Figure S3**

**
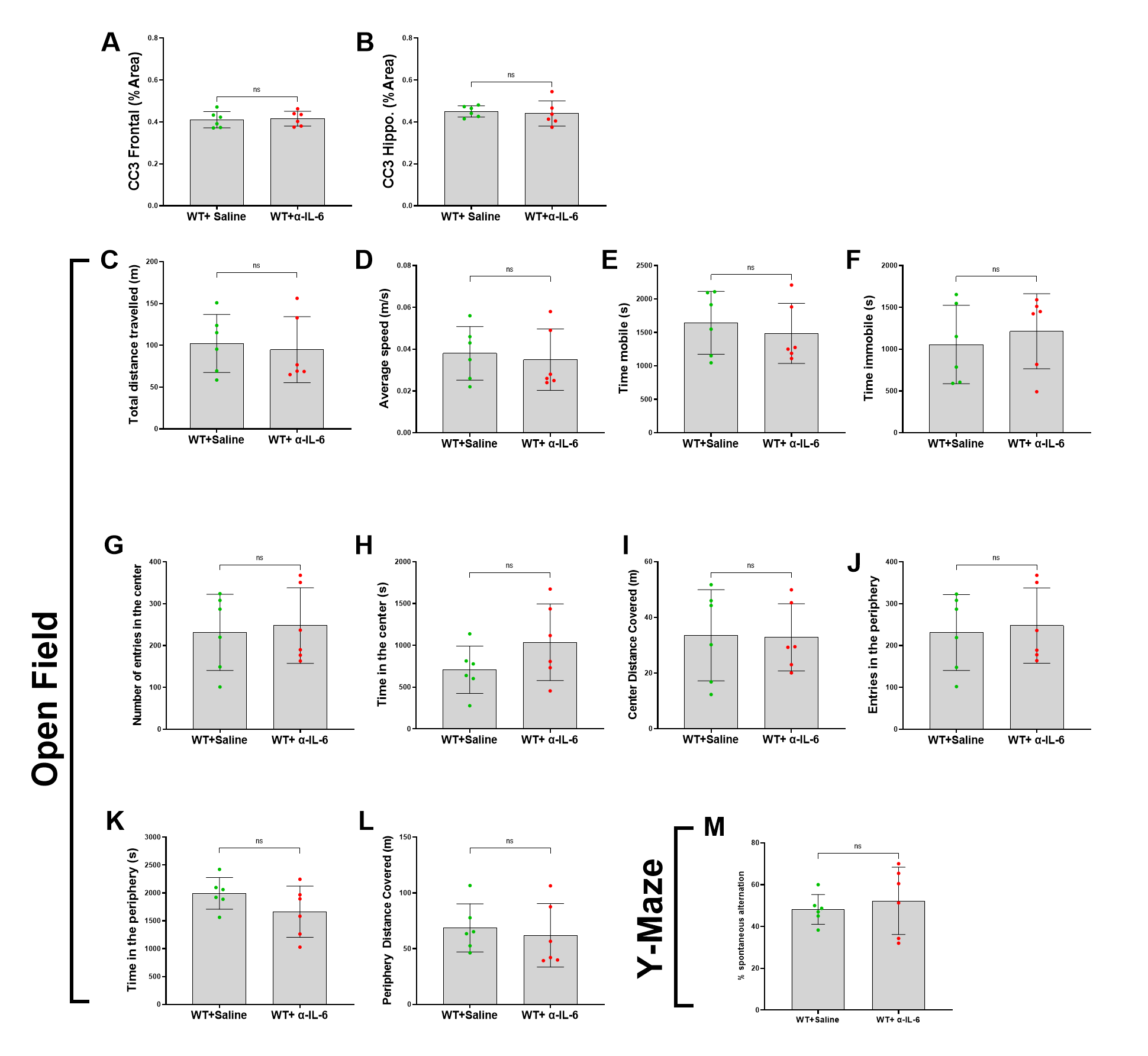
**

**α-IL-6 treatment to non-UTI mice causes no change in either brain histology or behavior.** Non-inoculated, wild-type mice were given the same α-IL6 antibody treatments as the UTI cohort, i.e., once daily doses for 3 days. A-B: IHC analysis of CC3 levels within the frontal cortex and hippocampus showed no difference in animals treated with the antibody compared to those treated with saline (vehicle). C-M: The mice underwent behavioral testing (i.e., open field and Y-Maze) and treated mice showed no difference in behavior compared to controls. Quantitative data are expressed in mean ± SD.

**Additional File, Figure S4**


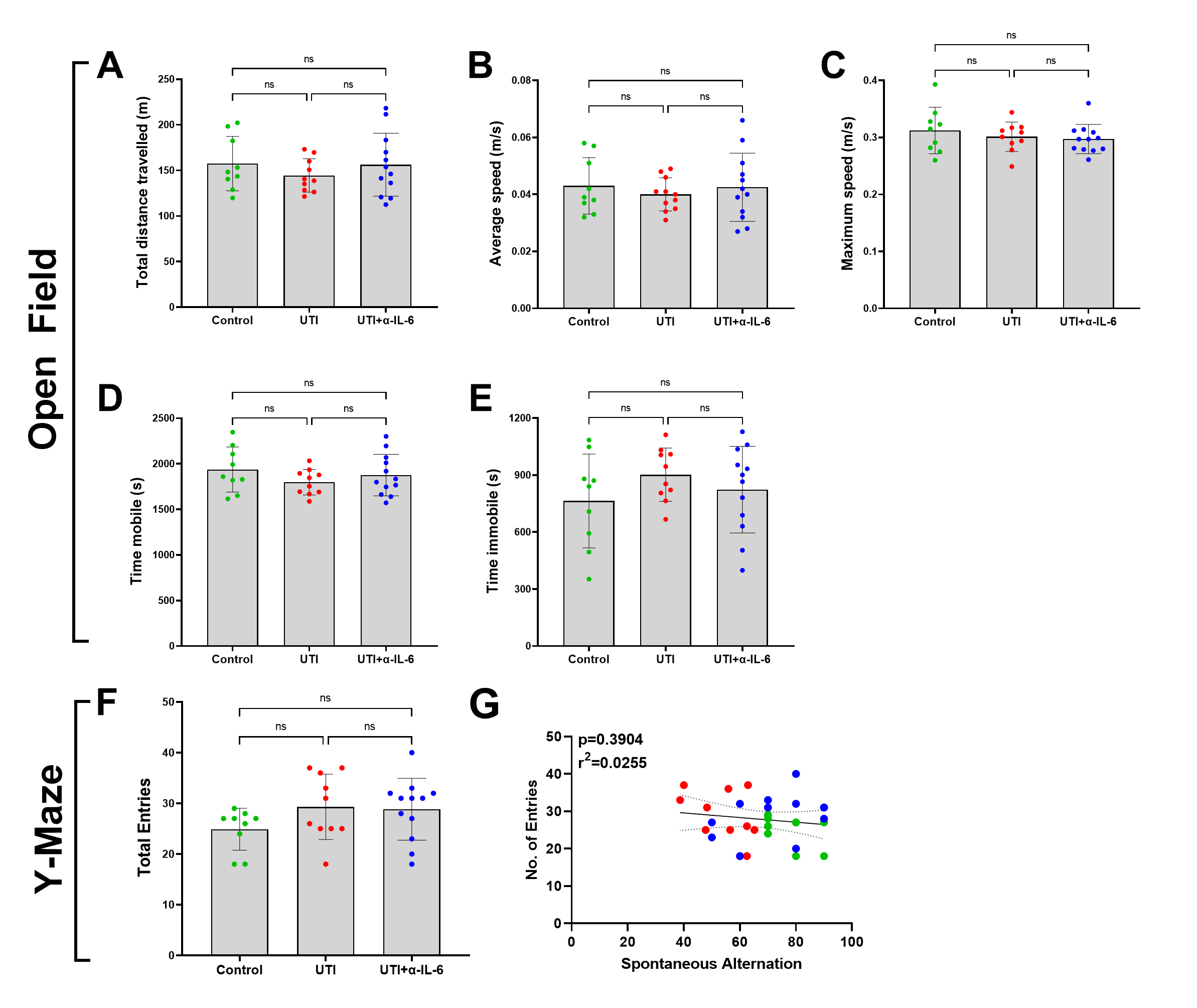


**There is no significant difference in overall locomotor activity in UTI compared to control or UTI+α-IL-6 groups.** **A-E:** Quantitative data of open field behavioral test show no significant difference in total distance traveled, average speed, maximum speed, total mobile time, or total immobile time between the groups. **F-G:** Quantitative data of Y-maze behavioral test showing no significant change in the total number of entries in different arms between the experimental groups. Each dot represents one animal (n=9 in control, n=10 in UTI, n=12 in UTI+α-IL-6).

**Additional File, Figure S5**


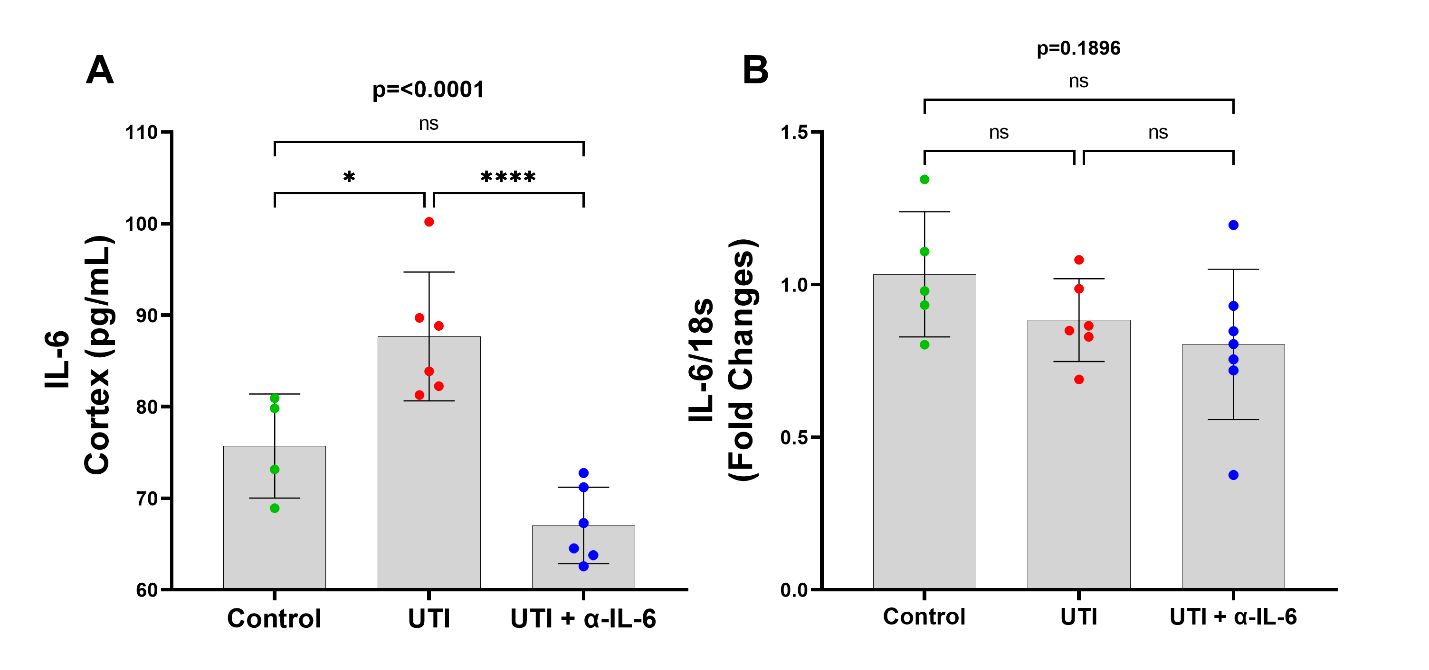


**A:** Quantitative data via ELISA corroborate immunohistochemistry findings by showing significant mitigation of frontal IL-6 in UTI mice treated with systemic anti-IL-6 antibody compared to UTI mice. **B:** Quantitative data via RT-PCR show no significant differences in cortical IL-6 mRNA across the three experimental groups. Quantitative data are expressed in mean ± SD. *p < 0.05, ****p < 0.0001.
